# Evaluating NG-Test CARBA 5 Multiplex Immunochromatographic and Cepheid Xpert CARBA-R Assays among Carbapenem-Resistant *Enterobacterales* Isolates Associated with Bloodstream Infection

**DOI:** 10.1128/spectrum.01728-21

**Published:** 2022-01-12

**Authors:** Yu-Tsung Huang, Yao-Wen Kuo, Nan-Yao Lee, Ni Tien, Chun-Hsing Liao, Lee-Jene Teng, Wen-Chien Ko, Po-Ren Hsueh

**Affiliations:** a Department of Laboratory Medicine, National Taiwan University Hospital, National Taiwan University College of Medicine, Taipei, Taiwan; b Department of Internal Medicine, National Taiwan University Hospital, National Taiwan University College of Medicine, Taipei, Taiwan; c Graduate Institute of Clinical Laboratory Sciences and Medical Biotechnology, National Taiwan University, Taipei, Taiwan; d Department of Integrated Diagnostics and Therapeutics, National Taiwan University Hospital, Taipei, Taiwan; e Department of Medicine, College of Medicine, National Cheng Kung University, Tainan, Taiwan; f Departments of Laboratory Medicine and Internal Medicine, China Medical University Hospital, School of Medicine, China Medical University, Taichung, Taiwan; g Colleague of Medicine, National Yang-Ming University, Taipei, Taiwan; h Department of Internal Medicine, Far Eastern Memorial Hospital, New Taipei City, Taiwan; I Ph.D Program for Aging, College of Medicine, China Medical University, Taichung, Taiwan; Houston Methodist Hospital

**Keywords:** carbapenem-resistant *Enterobacterales*, Xpert CARBA-R, NG-Test CARBA 5, multilocus sequence, *wzc*-genotyping, Xpert

## Abstract

Decreased susceptibility to carbapenems in *Enterobacterales* is an emerging concern. Conventional methods with short turnaround times are crucial for therapeutic decisions and infection control. In the current study, we used the Xpert CARBA-R (Cepheid, Sunnyvale, CA, USA) and the NG-Test CARBA 5 (NG Biotech, Guipry, France) assays for carbapenemase detection in 214 carbapenem-resistant *Enterobacterales* (CRE) blood isolates. We used the modified carbapenem inactivation method, conventional PCR, and sequencing to determine the production of five common carbapenemase families and their subtypes. We performed *wzc*-genotyping for all CR-Klebsiella pneumoniae (CRKP) and multilocus sequence typing for all carbapenemase-producing CRE isolates to reveal their genetic relatedness. The results showed a sensitivity of 99.8% and a specificity of 100% by the Xpert assay, and a sensitivity of 100% and a specificity of 99% by the NG-Test in detecting carbapenemases of 84 CRKP isolates with only one (VIM-1+IMP-8) failure in both tests. For CR-Escherichia coli, four carbapenemase-producing isolates were detected accurately for their subtypes. The two major clones of carbapenemase-producing CRKP isolates in Taiwan were ST11-K47 producing KPC-2 (*n *= 47) and ST11-K64 producing OXA-48-like (*n *= 9). Our results support the use of either test in routine laboratories for the rapid detection of common carbapenemases. Caution should be taken using the Xpert assay in areas with a high prevalence of CRE carrying *bla*_IMP-8_.

**IMPORTANCE** Carbapenemase-producing *Enterobacterales* (CPE) are emerging worldwide, causing nosocomial outbreaks and even community-acquired infections since their appearance 2 decades ago. Our previous national surveillance of CPE isolates in Taiwan identified five carbapenemase families (KPC, OXA, NDM, VIM, and IMP) with the KPC-2 and OXA-48-like types predominant. Timely detection and classification of carbapenemases in CPE may be a useful test to guide optimal therapy and infection control. Genetic detection methods using the Xpert CARBA-R assay and the immunochromatographic assay using the NG-Test CARBA 5 have been validated with the advantage of short turnaround time. Our study demonstrated that the NG and Xpert assays are convenient methods to accurately identify carbapenemases in carbapenem-resistant Klebsiella pneumoniae and carbapenem-resistant Escherichia coli blood isolates. Detecting IMP variants remains challenging, and the results of Xpert CARBA-R assay should be carefully interpreted.

## INTRODUCTION

Carbapenemase-producing *Enterobacterales* (CPE) are emerging in Taiwan, causing nosocomial outbreaks and even community-acquired infections since their appearance 2 decades ago (1–6). In our national surveillance of CPE isolates, we identified five carbapenemase families (KPC, OXA, NDM, VIM, and IMP) with the KPC-2 and OXA-48-like types predominant (2). A recent study revealed an increase in the carrying rate of metallo-beta-lactamase (MBL, e.g., *bla*_NDM_, *bla*_VIM_, and *bla*_IMP_) genes among carbapenem nonsusceptible Escherichia coli and Klebsiella pneumoniae isolates collected in Taiwan (5). Treatment options against CPE infections are limited, especially for isolates carrying MBL, which can hydrolyze novel beta-lactamase inhibitors (7–10). Commercial antimicrobial susceptibility tests for these new agents are not widely available in conventional laboratories; therefore, timely detection and classification of carbapenemases in CPE may be a useful alternative test to guide optimal therapy and infection control.

There are several methods for detecting carbapenemases, including phenotypic analyses of carbapenem hydrolyzation activity, molecular detection of specific genes, and immonochromatogenic assays for the enzymes (9). Complexity to perform, turnaround time, costs, and performance are important factors considered before applying these tests in routine laboratories. Genetic detection methods using the Xpert CARBA-R assay (Cepheid, Sunnyvale, CA, USA) and the immunochromatographic assay using the NG-Test CARBA 5 (NG Biotech, Guipry, France) have been validated with the advantage of short turnaround time (8, 9, 11). However, carbapenemase variants would affect the sensitivity of these methods (12, 13). Therefore, it is important to understand the local epidemiology of carbapenemases in CPE when evaluating these assays.

In this study, we compared the Xpert CARBA-R assay and the NG-Test CARBA 5 using CPE blood isolates collected from our previous national surveillance. We characterized the carbapenemases of the CPE isolates using a modified carbapenem inhibitory method and PCR (PCR) followed by sequencing of five carbapenemase genes (*bla*_KPC_, *bla*_OXA-48-like_, *bla*_NDM_, *bla*_VIM_, and *bla*_IMP_). We also determined the genetic relatedness of these CPE isolates by multilocus sequence typing (for E. coli and K. pneumoniae) and *wzc* gene polymorphism analysis (for K. pneumoniae) to determine whether clonal dissemination of isolates carrying specific carbapenemases occurred.

## RESULTS

We collected 186 CR-K. pneumoniae (CRKP) and 28 CR-E. coli (CREC) blood isolates from our analysis. There were 84 (45.2%) CRKP and 4 (14.3%) CREC isolates positive for the modified carbapenem inactivation method (mCIM), and all could be characterized into five major carbapenemases using reference PCR and sequencing (Table 1). Among CRKP, KPC was the most common carbapenemase (N = 66, 78.6%), followed by OXA (*n *= 10, 11.9%), VIM (*n *= 4, 4.8%), and NDM (*n *= 1, 1.2%). Three CRKP isolates had dual carbapenemases (OXA+KPC, OXA+NDM, and VIM+IMP). For the four CREC isolates, two each carried KPC and NDM.

**TABLE 1:** Comparison of the Xpert CARBA-R and the NG CARBA-5 tests with reference methods^*a*^

	Xpert CARBA-R						NG-Test CARBA 5					
Results by reference methods (*n*)^*b*^	TP	FP	FN	TN	Sensitivity (%)	Specificity (%)	TP	FP	FN	TN	Sensitivity (%)	Specificity (%)
Klebsiella pneumoniae (84)^*c*^												
KPC (67)	67	0	0	119			67	0	0	119		
OXA-48 like (12)	12	0	0	174			12	0	0	0		
VIM (5)	5	0	0	181			5	0	0	0		
NDM (2)	2	0	0	184			2	1	0	0		
IMP (1)	0	0	1	185			1	0	0	0		
Overall	83	0	1	102	99.8	100.0	83	1	0	102	100.0	99.0
Escherichia coli (4)												
KPC (2)	2	0	0	26			2	0	0	26		
NDM (2)	2	0	0	26			2	0	0	26		
Overall	4	0	0	24	100.0	100.0	4	0	0	24	100.0	100.0

a TP, true positive; FP, false positive; FN, false negative; TN, true negative

b A total of 186 carbapenem-resistant K. pneumoniae and 28 carbapenem-resistant E. coli isolates were enrolled for evaluation with 84 carbapenemases-producing K. pneumoniae and 4 carbapebnemases-producing E. coli identified by reference methods.

c Three isolates harbored more than one carbapenemase: one OXA+KPC, IMP+VIM, and OXA+NDM each.

The sensitivity and specificity of the two rapid tests for the five carbapenemases are summarized in Table 1. Only one isolate of CRKP carrying VIM+IMP, defined by the reference method, had discrepant results for both tests. The Xpert test was unable to identify the *bla*_IMP8_ variant, and the NG-Test found additional NDM enzymes that were not detected by the reference PCR. All three challenging CRKP isolates carrying *bla*_IMP8_ were also negative by the Xpert test but positive by the NG-Test, and each test was performed in duplicate.

The subtypes of carbapenemases, *wzc*-genotyping, and multilocus sequence typing (MLST) results for CRKP are summarized in Table 2. The most common carbapenemase subtypes in CRKP were KPC-2 (*n *= 56, 66.7%), followed by OXA-48-like (*n *= 9, 10.7%), KPC-17 (*n *= 8, 9.5%), VIM-1 (*n *= 4, 4.8%), KPC-3 (*n *= 2, 2.4%), NDM-1 (*n *= 1, 1.2%), and OXA-244 (*n *= 1, 1.2%). The subtypes of the three CRKP isolates carrying two carbapenemases were as follows: KPC-2+OXA-48-like, NDM-1+OCXA-48-like, and IMP-8+VIM-1. The carbapenemase subtypes of the four CREC isolates were diverse, including KPC-2, KPC-17, NDM-1, and NDM-5.

**TABLE 2:** Results of carbapenemases subtypes of Klebsiella pneumoniae isolates and their corresponding multilocus sequence type-capsular K type

Carbapenemases *(n)*	ST-capsular K type *(n)*
KPC2 (56)	ST11-K47 (47), ST11-Kna1^*a*^ (3), ST11-K64 (2), ST11-K3 (1), ST23-K47 (1), ST1460-K47 (1), ST2640-K2 (1)
KPC3 (2)	ST11-K47 (1), ST11-K54 (1)
KPC17 (8)	ST11-K47 (6), ST11-K54 (1), ST-1869-K47 (1)
OXA-48-like (9)	ST11-K64 (8), ST307-KN2 (1)
OXA-244 (1)	ST11-K64 (1)
VIM1 (4)	ST736-Knd^*b*^ (1), ST1947-K13 (1), STnew1^*c*^-K64 (1), STnew2^*c*^-K11 (1)
NDM1 (1)	ST15-K60 (1)
KPC2+OXA-48-like (1)	ST11-K64 (1)
OXA-48-like+NDM1 (1)	ST11-K64 (1)
VIM1+IMP8 (1)	ST8-K64 (1)

a Kna, sequences were not available by PCR.

b Knd, capsular serotype could not be determined by *wzc* gene analysis. (Refer to the Table S1 for the sequence).

c STnew, new sequence type.

A total of 23 genotypes were identified in 152 CRKP isolates by *wzc*-serotyping with K47 (*n *= 59, 38.8%) and K67 (N = 50, 32.9%) predominant. The distribution of capsular types differed significantly among isolates with and without carbapenemases (*P* < 0.001). Among the 84 CRKP isolates with carbapenemases, K47 (*n *= 58, 69.0%) was the most common genotype, followed by K64 (*n *= 14, 16.7%) (Table 2). Among the detected 102 CRKP isolates without carbapenemases, the most common capsular genotypes were K64 (N = 36, 35.3%), followed by KN2 (*n *= 8, 7.8%), K57 (*n *= 4, 3.9%), K2 (*n *= 3, 2.9%), K54 (*n *= 3, 2.9%), K1 (*n *= 2, 2.0%), K19 (*n *= 2, 2.0%), K24 (*n *= 2, 2.0%), and K62 (*n *= 2, 2.0%). Other capsular genotypes in non-carbapenemase CRKP included K5, K12, K14, K17, K21, K25, K27, K31, K38, and K47 (one case each). The results of sequence comparison are listed in the Table S2.

Results of MLST indicated that the most common sequence type (ST) was ST11 (N = 73; 86.9%) among carbapenemase-producing CRKPs. A difference of one allele was observed between ST1460 (*n *= 1) and ST1869 (*n *= 1) and ST11 and these were considered to be the same clonal complex 11. A difference in two alleles was found between ST23 (*n *= 1) and ST2640 (N = 1). Other STs included ST8, ST15, ST307, ST736, ST1947, and two new STs (Table 2). The allelic profiles are listed in the Table S3. There were no major STs among carbapenemase-producing CREC isolates ST354 (KPC-2), ST3492 (KPC-17), ST1193 (NDM-1), and ST410 (NDM-5). We observed geographic clustering of certain subtypes; seven out of eight KPC-17-producing CRKP isolates were isolated from a single institute in southern Taiwan, whereas nine out of the 12 OXA-48-like-producing CRKP isolates were from central Taiwan.

## DISCUSSION

Our results showed excellent performance of both rapid tests for detecting major carbapenemase variants (KPCs and OXA-48-like) of CRE in Taiwan (sensitivity and specificity >99%). Clonal outbreaks of ST11-K47 and ST11-K64 comprised the two major carbapenemase-producing CRKP clones in our surveillance. Among non-carbapenemase-producing CRKP, ST11-K64 was predominant.

Both the NG-Test and Xpert assay can shorten the overall turnaround time to within 2 h to detect and differentiate types of carbapenemases accurately from either bacterial colonies or directly from clinical samples (8, 14–17). Based on the prevalence of CRE carrying carbapenemases in Taiwan (41.2%), the positive predictive value and the negative predictive value would be 100.0% and 99.2% (95% confidence interval: 94.7%–99.9%) for the Xpert assay and 98.9% (95% C.I.: 92.7%–99.8%) and 100% for the NG test (6).

The accuracy of the two tests is affected by variants of carbapenemases, especially false-negative results of both tests for detecting IMP (IMP-13/IMP-15/IMP-18 clades) and OXA families (13, 18–20). Recent studies enrolled isolates carrying KPC, OXA-48 VIM and NDM variants, and only few IMP variants (IMP-4 and IMP-6) were evaluated (17, 21, 22). Contrary to our study, Liu showed that the NG test missed 8 NDM, 4 OXA-48 and 1 IMP blood isolates, especially when co-carriage of carbapenemases compared with the Xpert assay (21). However, they did not specify the variants of the carbapenemases resulting in false-negative by the NG test. Our results showed that *bla*_IMP-8_ in CRKP may not be detected accurately by the Xpert assay. Similarly, Jenkins et al. reported two Enterobacter cloacae and one Serratia marcescens isolate carrying *bla*_IMP8_ that had discrepant results with false-negatives by the Xpert assay and remaining positive by the NG-Test (13). The IMP-8-producing *Enterobacterales* have caused several outbreaks in Taiwan since the first report in 2001 (3, 23). Tseng et al. reported that among 183 carbapenemase-producing K. pneumoniae isolates, IMP-8 was the second most common carbapenemase (8.7%) after KPC (24). In a recent surveillance, IMP-8 remained a significant part of carbapenemase-producing E. coli (30.4%) and K. pneumoniae (4.6%) isolates collected during 2016–2018 (5). There was no clustering of IMP-8-producing CRKP for the identified MLSTs, including ST37, ST76, ST255, and ST919 (5). Detection of the IMP family remains challenging using PCR-based methods owing to the even distribution of mutations along the diversified sequences of different variants (18, 20). Other rapid tests, such as the CARBA NP test, utilizes the ability of carbapenemase hydrolysis of carbapenem for detection. However, the sensitivity (77.4%–90.0%) and specificity (85.7%–100.0%) varied and may differ among laboratories (15). Baeza et al. proposed a new algorithm using an immunochromatographic assay followed by an mCIM test (zCIM) to avoid false negatives of rare carbapenemases and shorten turnaround time in routine laboratories (8). Clinicians and technicians should be cautious in interpreting negative results by the Xpert assay in IMP-8 areas of endemicity and may incorporate the workflow suggested to avoid false results by these rapid tests.

Decreased susceptibility of carbapenem against *Enterobacterales* in Taiwan is an important issue, especially the dissemination of carbapenemase-producing K. pneumoniae in the last decade. Up to 29%–47% of CRKP isolates carried carbapenemase in several surveillances for resistance compared to less than 10% before 2011 (6). The KPC-2-producing ST11-K47 and OXA-48-producing ST11-K64 CRKPs are the two predominant carbapenemase-producing clones identified and some isolates among these clones also carry several virulence genes associated with community onset invasive syndromes in Asia (1, 25–28). We identified several serotypes and ST types that have been associated with the hypervirulent strains in our CRKP blood isolates. Although rarely isolated from patients with invasive syndrome currently, monitoring of these carbapenemase-producing clones and further investigation of virulence in these isolates are warranted.

The predominant mechanisms of CREC in Taiwan are the loss of CMY-2 AmpC beta-lactamases combined with porin (OmpF/OmpC) loss, and the emergence in recent years of carbapenemase-producing CREC, ranging from 7.6% to 29.5% of isolates studied (5, 29). The NDM families (NDM-1,5) were the most common carbapenemases carried in E. coli because of clonal spread of ST410, which was also detected in one of our isolates (5). Other carbapenemases in E. coli, including KPC-2, IMP-8, VIM-1, OXA-48, and OXA-131, were found in earlier resistance surveillances (5, 6, 29). Timely detection using rapid tests and imposing strict infection control would mitigate the spread of these multiple drug-resistant clones.

Our study has several limitations. First, we did not perform sequence typing for non-carbapenemase-producing CRKP, and it is not clear if clonal dissemination occurred in the predominant K64 CRKP. Second, we used primers designed for commonly encountered carbapenemases in our reference PCR method and these may be unable to detect rare variants. The isolate positive for three carbapenemases by the NG-Test should be examined using the whole-genome sequence to exclude the possibility of a new NDM variant. Finally, except for KPC and OXA-48-like, we collected a few isolates from other carbapenemase families in our experiment. The performance of the two tests for detecting the other three carbapenemase families in Taiwan requires further investigation.

In conclusion, the NG and Xpert assays are convenient methods to accurately identify carbapenemases in CRKP and CREC blood isolates. Detecting IMP variants remains challenging, and the results of PCR-based rapid tests should be carefully interpreted. The protein-level detection of the NG test is more adapted to reduced variation caused by molecular modifications. We found clonal dissemination of CRKP isolates carrying *bla*_KPC-2_ and *bla*_OXA-48-like_ genes in Taiwan and geographic variation in major carbapenemase types. Through combining these rapid tests in the workflow of routine laboratories facing CRE, clinicians could prescribe suitable antibiotics against these organisms and be aware of the emergence of MBLs among CRE isolates.

## MATERIALS AND METHODS

### Bacterial isolates.

We selected blood isolates of CRKP and CREC, collected during 2017–2019 from the Surveillance of Multicenter Antimicrobial Resistance in Taiwan program conducted by the Taiwan Centers for Disease Control (30). *Enterobacterales* isolates resistant to either imipenem, meropenem, or ertapenem were considered carbapenem-resistant. These isolates were analyzed in our previous studies (2, 30, 31). Three CRKP isolates carrying *bla*_IMP-8_ from a previous outbreak were enrolled to verify our final findings (3). Species were reconfirmed by matrix-assisted laser desorption ionization–time of flight mass spectrometry, and isolates were stored at −80°C with 20% glycerol before testing. The institutional review board of the National Taiwan University Hospital waived the need for written informed consent because the study involved only a minimal risk to the patients (201609066RINB).

### Modified carbapenem-inhibitory methods.

We performed mCIM for all isolates, and the results were interpreted according to the recommendations of the Clinical and Laboratory Standards Institute guidelines (32). K. pneumoniae ATCC BAA1705 and K. pneumoniae ATCC BAA1706 were used as positive and negative-control strains, respectively.

### Determination of carbapenemase-encoding genes in K. pneumoniae and E. coli isolates.

Bacterial isolates were cultured overnight on blood plates overnight before testing. We performed PCR and bidirectional sequencing for all CRE isolates using primers and PCR conditions described elsewhere to detect five common carbapenemase families (*bla*_KPC_, *bla*_OXA-48-like_, *bla*_NDM_, *bla*_VIM_, and *bla*_IMP_) (1, 2). The results of sequencing were used as references to determine the sensitivity and specificity of the two commercial methods. We performed the Xpert CARBA-R assay (Cepheid, Sunnyvale, CA, USA) and an immunochromatographic assay using the NG-Test CARBA 5 (NG Biotech, Guipry, France) for all CRE isolates according to the manufacturer’s instructions. In case of discrepant results, we duplicated the experiments.

### Serotyping of carbapenemase-producing K. pneumoniae and multilocus sequence typing for CPE.

We performed serotyping by *wzc* gene polymorphism analysis for all CRKP isolates and determined the MLST for all carbapenemase-producing CRKP and CREC isolates as previously described. In brief, the *wzc* gene was PCR-amplified and sequenced as described by Pan et al. (33). Regarding MLST, seven (*gapA*, *inf*, *mdh*, *pgi*, *phoE*, *rpoB*, and *tonB*) and eight (*dinB*, *icdA*, *pabB*, *polB*, *putP*, *trpA*, *trpB*, and *uidA*) housekeeping genes were sequenced for K. pneumoniae and E. coli, respectively. The allele naming and ST identification were assigned according to the database at www.pasteur.fr/mlst.

### Data analysis and statistics.

The sensitivity and specificity of the Xpert assay and NG-Test were calculated. To compare the capsular serotype of CRKP with or without carbapenemases, we used the chi-square test. Statistical significance was set at *P* < 0.05. Data were analyzed using STATA software (version 14.0; StataCorp., College Station, TX, USA).
