# Supplementary material for: Evaluating NG-Test CARBA 5 Multiplex Immunochromatographic and Cepheid Xpert CARBA-R Assays among Carbapenem-Resistant Enterobacterales Isolates Associated with Bloodstream Infection
Source: Microbiol Spectr. 2022 Jan 12;10(1):e01728-21. doi: 10.1128/spectrum.01728-21 (PMC8754146; doi:10.1128/spectrum.01728-21)
Supplement: SUPPLEMENTAL FILE 1 — Supplemental material. Download SPECTRUM01728-21_Supp_1_seq4.pdf, PDF file, 0.5 MB [file spectrum01728-21_supp_1_seq4.pdf]

Supplementary TABLE 1. The CD1-VR2-CD2 region of *wzc* gene sequences of the new K type. The closet K-type is K12.

Locus:	*wzc* (CD1-VR2-CD2 region of *wzc*)
Allele	13
Sequence	GCTTTGTTATTGATTTCTCATTGCGATATCCGCAGGATTCTCAACTGCAAGAAACCCTTGATTCTCATCATTTCCTTTGTTTTTCCAGTTA<br>GATTTTTTCGTAACCTTTTGAGAGAATGTTTCAGCAACAGGAATACTTGCATAAACAGTTATGCCAATTTCCTCAAGTTGCTCAGGACTTT<br>CGATACCGCGTCGTAAAAATATGCGTAGTAATACTAAACCTATTGAGACTAAAACACCTAAAAATACGCCAATCAATACCACAGCAATTTT<br>TTTCGGTTTTACCGGCTTAGGTTGCGTTACAGCATTATCTATAATACGTACATTCCCTATAGCACTCGATTTTGCAATACTCAATTCTTGCT<br>GACGATTCAGTAACTGCATATATACTGCCCGGCCAGACTCGACATCTCTACTTAAGCGCAATATTTCTTGCTGGGTTTCAGGCATAGTTG<br>AAACGCGTTTATTCAATTTTGTTTTTTCATCTTGGAGTGTTTTACGTTTCTCCAGCAAAGCTTTATAGGTAGGATGCTCTTTCGTGTAAAG<br>TTGCGAAATTTCAGACTCACGGAATGTTAGCTCATTCAACTGATTATCAACATTGACAATTTGATCCAGTACAGATTTTGCTTCTAATGAC<br>AAATCTACCGAATCATTCTGACGGCGATATGCATTCAGATTATTCTCAGCTGAATCTAAGCTATCTCTCACTTTTGGCAATTGTTGGTTTA<br>AAAACTCTAAACTTTTTTGATCTTGGGCAGCCTGCCGTGCTATGTTTTGGGCAAGATATCCTTCACTAATATTGTCAATAATTTCTTTTATT<br>AATACAGGATTATCACCAGTGACCGTGAGTGTAAGTATACCGGTATCTTTGCCTTGATCGGCAACCGAAAATACTTGTTGTAAATCTGTT<br>ATTGCTTTCAAACGAGTTACTGATGAAATATTAAATGTCGTTCCCGCTGGAGCATCTATAGAGTCAACTTTTATAGAAATTCCATGCCTTC<br>AACTAGTTCACCAACAGCTCCTTTAAGTCAAAGTCATCTCTGATATACGTAGTGCGAAGAGTCTATAACGTTTATTTTAGCTTAACTACATT<br>CTTCGTCTCCATTAACTGCCA

Supplementary TABLE 2. The results of sequence comparison of *wzc* gene CD1-VR2-CD2 region for carbapenem-resistant *K. pneumoniae* isolates. ND: new sequence types.

Supplementary TABLE 3. The allelic profiles of carbapenemases-producing carbapenem-resistant *Klebsiella pneumoniae* isolates in current study.

**Sequence type**	***gapA***	***infB***	***mdh***	***pgi***	***phoE***	***rpoB***	***tonB***
8	4	1	1	1	1	5	6
11	3	3	1	1	1	1	4
15	1	1	1	1	1	1	1
23	2	1	1	1	9	4	12
307	4	1	2	52	1	1	7
412	2	1	2	1	9	1	112
736	42	22	55	22	11	13	167
1460	4	3	1	1	1	1	4
1869	3	3	1	1	9	1	4
1947	16	24	21	143	47	60	67
2640	2	1	1	1	1	4	13
STnew1	269	19	79	20	113	21	153
STnew2[a]	98	19	90	20	117	21	162

[a] One allele (*gapA*) differs from ST2298.
